# Prevention of Decay

**Published:** 1900-03-15

**Authors:** J. Leon Williams


Prevention of Decay.—In my own practice I rely chiefly upon a strong solution of hydronapthol in oil of cassia. This I use freely in all cavities, and before filling I use a varnish of Canada balsam in chloroform in which there is 10 percent of hydronapthol.—*J. Leon Williams, Items of Interest.*

